# Draft Genome Sequence of *Enterococcus faecalis* Strain UCD-PD3

**DOI:** 10.1128/genomeA.01386-16

**Published:** 2016-12-15

**Authors:** Dana R. De Vries, Alexandra L. Martin, Holly H. Ganz, Jonathan A. Eisen, David A. Coil

**Affiliations:** a Davis Genome Center, University of California Davis, Davis, California, USA; b Department of English Literature, University of California Berkeley, Berkeley, California, USA; c Department of Evolution and Ecology and Department of Medical Microbiology and Immunology, University of California Davis, Davis, California, USA

## Abstract

Here, we present the draft genome sequence of *Enterococcus faecalis* strain UCD-PD3. The assembly contains 2,861,314 bp in 73 contigs. This strain was isolated from a feral domestic cat (*Felis catus*) anal sac secretion sample, as part of a project on isolating and characterizing the microbes present in feline anal sacs.

## GENOME ANNOUNCEMENT

*Enterococcus faecalis* is commonly found in the gut of mammals and is known to be a symbiotic bacterium (1). *E. faecalis* UCD-PD3 was isolated from feline anal sac secretions collected as a part of a larger study of the microbiology of cats (kittybiome). Here, the goal was to isolate and characterize bacterial isolates from anal sacs from the domestic cat *Felis catus*. Anal sacs were expressed as a part of a spay and neuter clinic on feral cats. The anal sac is considered an anaerobic environment, capable of supporting the growth of *E. faecalis*, an aerotolerant anaerobe (2). Swab samples of the secretions were collected and placed in 1× phosphate-buffered saline (PBS). We inoculated 50 µl of diluted anal sac secretion onto Colombia blood agar and incubated at 37°C for 5 days under low-oxygen conditions (BD GasPak EZ container system). One colony was selected and subcultured onto Colombia blood agar and streaked for isolation. A fresh colony was subcultured three times and incubated under the same conditions for 5 days each time. DNA was then extracted directly from an isolated colony using a Promega Wizard genomic DNA purification kit. PCR was performed to amplify the 16S rRNA gene using 27F and 1391R primers. The PCR product was sequenced using Sanger sequencing, and the consensus sequence was identified using BLAST (3). Using the Ribosomal Database Project (RDP), an alignment was created between this isolate and other *Enterococcus* species isolates (4). An approximate maximum likelihood phylogenetic tree was created in FastTree and viewed in Dendroscope (5, 6). This isolate was found in a clade containing other *Enterococcus faecalis* strains.

A paired-end library was created using a Nextera XT library preparation kit (Illumina) in preparation for whole-genome sequencing. Using a PippinPrep (Sage Science), we selected 600- to 900-bp fragments. The size-selected library was sequenced on a paired-end 300-bp run of an Illumina MiSeq. Following the completion of quality trimming and error correction by the A5-miseq assembly pipeline, 807,883 high-quality reads were assembled into 73 contigs, with 36× coverage and a G+C content of 37.6% (7, 8). Genome completeness was estimated using PhyloSift software, which searched for 37 highly conserved single-copy marker genes, and one copy of each was found in this assembly (9).

Annotation was performed using RAST (10). *E. faecalis* strain UCD-PD3 contains 2,686 predicted coding sequences and 59 noncoding RNAs. The full-length 16S rRNA sequence (1,552 bp) was analyzed using BLAST and matched with 100% identity with other *E. faecalis* strains.

### Accession number(s).

This whole-genome shotgun project has been deposited at DDBJ/ENA/GenBank under the accession no. LYBN00000000. The version described in this paper is version LYBN01000000.
